# Increased amyloidogenic APP processing in *APOE* ɛ4-negative individuals with cerebral β-amyloidosis

**DOI:** 10.1038/ncomms10918

**Published:** 2016-03-07

**Authors:** Niklas Mattsson, Philip S. Insel, Sebastian Palmqvist, Erik Stomrud, Danielle van Westen, Lennart Minthon, Henrik Zetterberg, Kaj Blennow, Oskar Hansson

**Affiliations:** 1 Clinical Memory Research Unit, Department of Clinical Sciences Malmö, Lund University, Simrisbanvägen 14, Malmö 212 24, Sweden; 2 Memory Clinic, Skåne University Hospital, Simrisbanvägen 14, Malmö 212 24, Sweden; 3 Department of Neurology, Skåne University Hospital, Getingevägen 4, Lund 222 41, Sweden; 4 Center for Imaging of Neurodegenerative Diseases, Department of Radiology and Biomedical Imaging, San Francisco Veterans Affairs Medical Center (SFVAMC) campus, 4150 Clement Street, University of California, San Francisco, California 94121, USA; 5 Department of Clinical Sciences, Diagnostic Radiology, Lund University, Box 117, Lund 221 00, Sweden; 6 Center for Medical Imaging and Physiology, Skåne University Hospital, Getingevägen 4, Lund 222 41, Sweden; 7 Department of Molecular Neuroscience, UCL Institute of Neurology, Queen Square, London WC1N 3BG, UK; 8 Clinical Neurochemistry Laboratory, Institute of Neuroscience and Physiology, The Sahlgrenska Academy at University of Gothenburg, Mölndal 431 80, Sweden

## Abstract

Increased APP (amyloid precursor protein) processing causes β-amyloid (Aβ) accumulation in autosomal dominant Alzheimer's disease (AD), but it is unclear if it also affects sporadic Aβ accumulation. We tested healthy controls and patients with mild cognitive symptoms (*N*=331) in the BioFINDER study, using cerebrospinal fluid (CSF) Aβ40 as a surrogate for amyloidogenic APP processing. We find that levels of brain Aβ fibrils (measured by 18F-flutemetamol PET) are independently associated with high CSF Aβ40 (*P*<0.001) and *APOE* ɛ4 (*P*<0.001). The association between CSF Aβ40 and brain Aβ is stronger in *APOE* ɛ4-negative than in positive people (*P*=0.0080). The results are similar for CSF Aβ38 and for a combination of CSF Aβ38 and CSF Aβ40. In conclusion, sporadic Aβ accumulation may be partly associated with increased amyloidogenic APP production, especially in *APOE* ɛ4-negative subjects. The risk for sporadic AD may consequently depend on increased Aβ production, in addition to decreased Aβ clearance.

## Abstract (web summary)

Autosomal dominant Alzheimer's disease is thought to be caused by increased amyloidogenic APP processing. Mattson *et al.* show that association between brain Aβ and cerobrospinal fluid Aβ40 levels is stronger in APOE ɛ4 negative people, suggesting that increased processing may also underlie sporadic disease.

## Graphical abstract

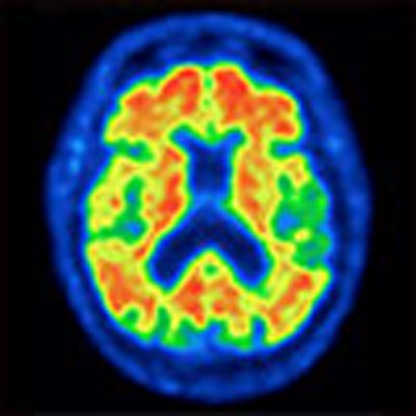

Brain accumulation of amyloid β (Aβ) is a hallmark of Alzheimer's disease (AD) which may precede dementia by up to two decades^1^,^2^,^3^ and be quantified by cerebrospinal fluid (CSF) biomarkers or positron emission tomography (PET) imaging^4^,^5^. Aβ accumulation is thought to be caused by an imbalance of Aβ production and clearance from the brain^6^. The *APOE* ɛ4 allele is the main genetic susceptibility factor for late-onset AD and sporadic Aβ pathology^7^. This is likely because the *APOE* ɛ4 gene product apoE4 has reduced capacity to clear Aβ peptides from the brain^8^. However, Aβ accumulation also occurs in the absence of *APOE* ɛ4 (ref. 7) and ∼40–50% of AD patients lack the *APOE* ɛ4 allele^9^. In autosomal dominant forms of AD, Aβ pathology is believed to be caused by increased amyloidogenic processing of APP (amyloid precursor protein), that is, increased Aβ production^10^ but variations in APP processing have not been thoroughly explored as risk factors in ‘sporadic' AD. Using a large cohort of non-demented subjects, the aim of this study was to test if *APOE* ɛ4 and biomarker surrogates of amyloidogenic APP processing were independently associated with brain Aβ accumulation. We used CSF levels of Aβ40 to estimate amyloidogenic APP processing. The rationale for this was that Aβ40 is a major Aβ isoforms produced by neurons by concerted β- and γ-secretase cleavages of APP (the same processing pathway that results in Aβ42)^11^ but is generally not related to Aβ plaque pathology (in contrast to CSF Aβ42, which is reduced in the presence of Aβ plaques^12^). Note that previous studies testing the correlation between CSF Aβ40 and PET Aβ have not co-varied for the presence of *APOE* ɛ4. We hypothesized that there would be independent correlations between Aβ accumulation and the predictors *APOE* ɛ4 and CSF Aβ40, and that increased amyloidogenic APP processing would be related to Aβ accumulation mainly in *APOE* ɛ4-negative subjects. We also hypothesized that CSF Aβ40 would not be associated with *APOE* ɛ4 (that is, CSF Aβ40 would not be affected by apoE4-mediated impaired Aβ clearance). Finally, we hypothesized to see similar results when using CSF Aβ38 instead of CSF Aβ40 to estimate amyloidogenic APP processing.

Our results confirmed our hypothesis. We show that 18F-flutemetamol PET levels are independently associated with high CSF Aβ40 (*P*<0.001) and *APOE* ɛ4 (*P*<0.001) and that the association between CSF Aβ40 and brain Aβ is stronger in *APOE* ɛ4-negative than in positive people (*P*=0.0080). The results are similar when using CSF Aβ38 or a combination of CSF Aβ38 and CSF Aβ40 to estimate amyloidogenic APP production. We conclude that sporadic Aβ accumulation may be partly associated with increased amyloidogenic APP production, especially in *APOE* ɛ4-negative subjects. Thus, the risk for sporadic AD may partly depend on increased Aβ production, in addition to decreased Aβ clearance.

## Results

### Cohort characteristics

**Figure 1:**
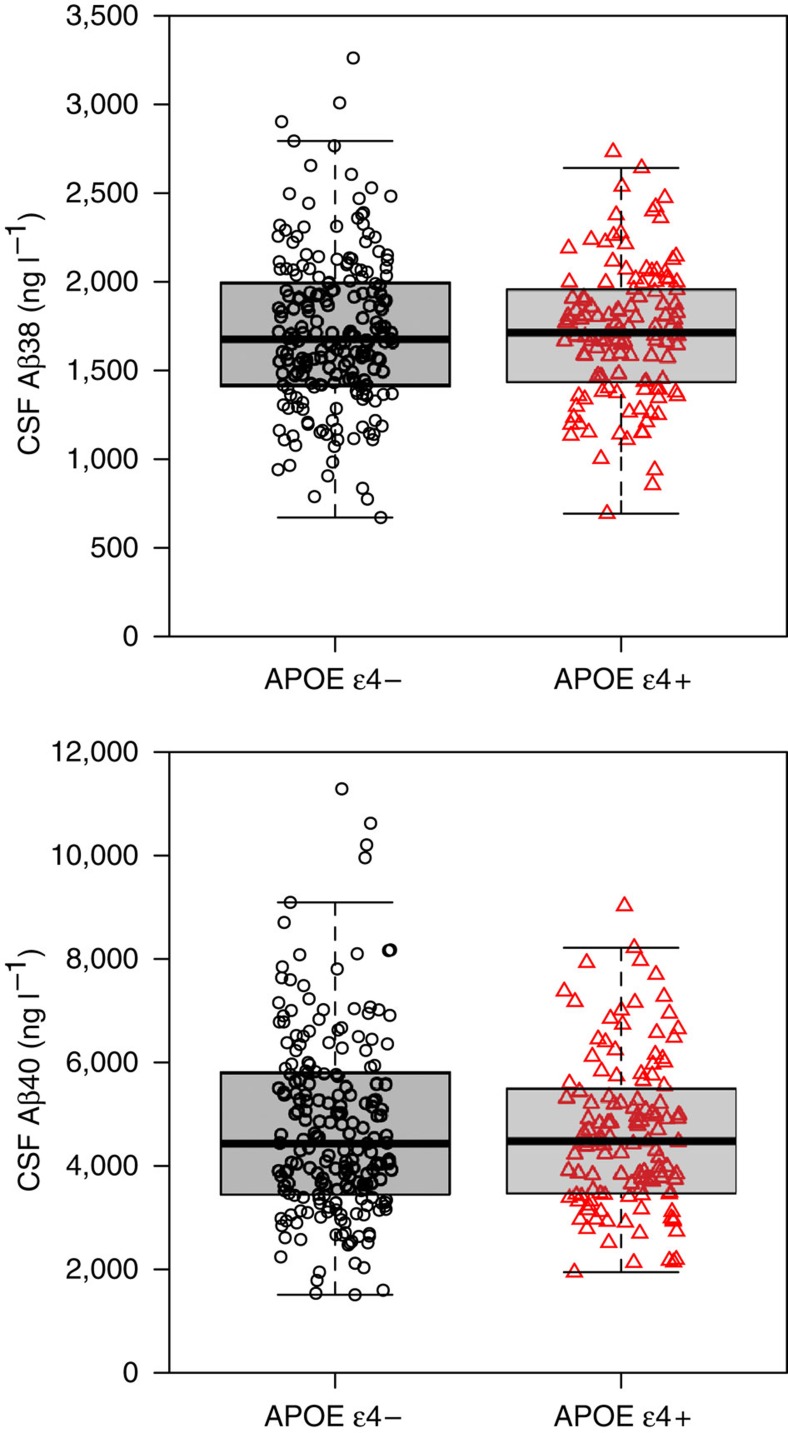
CSF Aβ38 and Aβ40 in *APOE* ɛ4- and *APOE* ɛ4+ subjects. Observed data for CSF Aβ38 and CSF Aβ40 by *APOE* ɛ4 status. The individual observations are overlaid on boxplots (thick lines are medians, box limits are 25th and 75th percentiles). *APOE* ɛ4 did not affect levels of CSF Aβ38 (Mann–Whitney U-test, *P*=0.75; *t*-test, *P*=0.95; linear regression adjusted for age, sex and diagnosis, *P*=0.99) or CSF Aβ40 (Mann–Whitney U-test, *P*=0.84; *t*-test, *P*=0.43; linear regression adjusted for age, sex and diagnosis, *P*=0.26). This supports the notion that CSF Aβ38 and Aβ40 are unaffected by *APOE* ɛ4-mediated changes in Aβ clearance.

The cohort consisted of 331 participants (cognitively normal controls (CN) 121, subjective cognitive decline (SCD) 102 and mild cognitive impairment (MCI) 108). Demographics and data on cognition and biomarkers are summarized in Table 1 (see Table 2 for demographics stratified by *APOE* status). In sum, *APOE* ɛ4 positivity was more common in SCD and MCI than in CN, CSF Aβ42 levels were lower in MCI compared with the other groups, and the frequency of PET Aβ positivity was lowest in CN and highest in MCI. CSF Aβ38 and CSF Aβ40 did not differ between the diagnostic groups. *APOE* ɛ4 was not associated with CSF Aβ40 or with CSF Aβ38 (Fig. 1). The lack of association between *APOE* ɛ4 and CSF Aβ40 and CSF Aβ38 supports our assumption that these CSF Aβ peptides are unaffected by apoE4-mediated clearance of Aβ.

### APOE ɛ4 and high CSF Aβ40 independently predict PET Aβ

**Figure 2:**
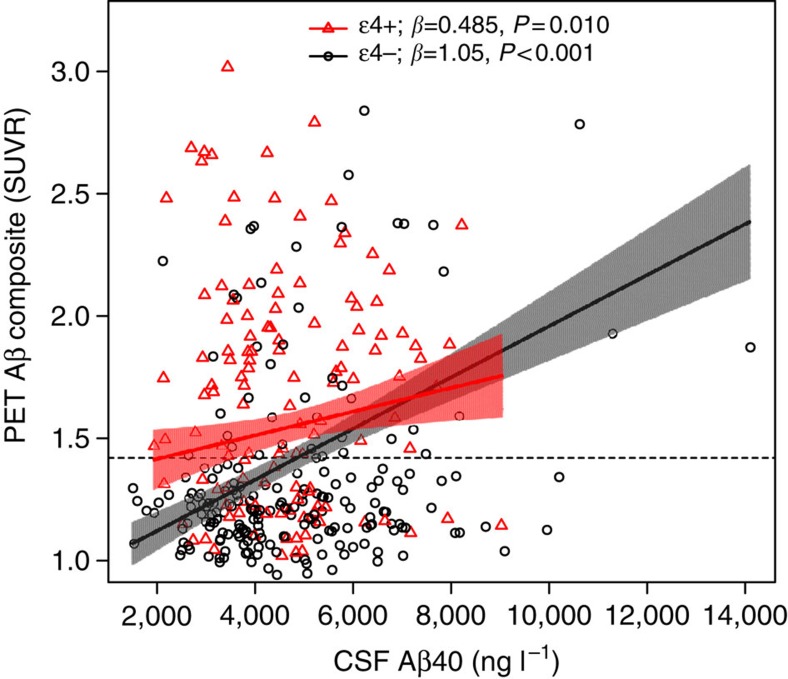
PET Aβ as a function of CSF Aβ40 and *APOE* ɛ4. Observed PET and CSF Aβ40 data. Slopes are modelled from a linear regression adjusted for CSF Aβ42, sex, age and diagnostic group. The shaded areas indicate 95% confidence intervals for the slopes. The dotted line indicates a cutoff for clinically significant PET Aβ load (1.42 SUVR). β-coefficients (divided by 10^−4^) and *P* values for the slopes within *APOE* ɛ4-positive and separately for *APOE* ɛ4-negative subjects are shown in the legend. The interaction between CSF Aβ40 and *APOE* ɛ4 is significant, indicating that the correlation between CSF Aβ40 and PET Aβ differs by *APOE* ɛ4 status (*P*=0.0080). The results did not change significantly when removing outliers (CSF Aβ40>10,000 ng l^−1^).

Figure 2 shows the observed PET Aβ and CSF Aβ40 data, with estimated slopes in the *APOE* ɛ4-positive and -negative groups. In a linear regression model with PET Aβ as the dependent variable, high levels of CSF Aβ40 (*β*=1.05 × 10^−4^, *P*<0.001), *APOE* ɛ4-positivity (*β*=0.406, *P*<0.001) and the interaction between *APOE* ɛ4 and CSF Aβ40 (β=−5.61 × 10^−5^, *P*=0.0080) were all significant predictors of continuous PET Aβ. Note that since *APOE* ɛ4 and CSF Aβ40 were both included as predictors the main effect of CSF Aβ40 indicates the effect within *APOE* ɛ4-negative subjects. The significant interaction between CSF Aβ40 and *APOE* ɛ4 indicates that the correlation between CSF Aβ40 and brain Aβ was stronger in *APOE* ɛ4-negative than in positive people (as seen in Fig. 2). The correlation between CSF Aβ40 and PET Aβ in the *APOE* ɛ4-positive group was weaker than in the *APOE* ɛ4-negative group, but remained significant (*β*=0.485 × 10^−4^, *P*=0.010). The results support the hypotheses that high CSF Aβ40 and *APOE* ɛ4 are independent predictors of PET Aβ, and that the relationship between CSF Aβ40 and PET Aβ varies with *APOE* ɛ4 carrier status. As expected, CSF Aβ42 was a significant covariate (low CSF Aβ42 was correlated with PET Aβ, *β*=−0.00120, *P*<0.001), but CSF Aβ40, *APOE* ɛ4 and the interaction between CSF Aβ40 and *APOE* ɛ4 remained significant also when not adjusting for CSF Aβ42 (CSF Aβ40: *P*=0.0089; *APOE* ɛ4: *P*<0.001; interaction: *P*=0.026). Age (*β*=0.0082, *P*=0.0094) and diagnosis (SCD, *β*=0.137, *P*=0.0011; MCI, *β*=0.299, *P*<0.001) were also significant predictors of PET Aβ, but sex was not (*P*=0.23). White matter lesions (WML) were evaluated as a covariate, but were not significant (*P*=0.68) and were therefore excluded from the final model. We also evaluated plasma levels of Aβ40 as a covariate to exclude the possibility that the results depended on peripheral APP processing. Plasma Aβ40 was not a significant covariate (*P*=0.99) and including it in the model did not change the other estimates.

To further examine if clinically significant Aβ accumulation (defined as a composite standardized uptake value ratio (SUVR) >1.42 (ref. 13) was associated with CSF Aβ40, we evaluated a logistic regression model with PET Aβ positivity as the dependent variable. CSF Aβ40 (log odds=9.18 × 10^−4^, *P*<0.001) was a significant predictor in this model but *APOE* ɛ4 (*P*=0.13), and the interaction between CSF Aβ40 and *APOE* ɛ4 were not (*P*=0.73). Age and sex (*P*=0.13–0.14) were not significant but CSF Aβ42 (*P*<0.001) and diagnosis (SCD, *P*=0.0016; MCI, *P*<0.001) remained significant covariates. When CSF Aβ42 was excluded from the model, CSF Aβ40 (*P*=0.014) and *APOE* ɛ4 (*P*<0.001) were both significant predictors of PET Aβ positivity.

### CSF Aβ38 as an independent predictor of PET Aβ

To corroborate our findings, we repeated the analyses using CSF Aβ38 instead of CSF Aβ40, with very similar results. When predicting continuous PET Aβ, the effects of CSF Aβ38 (*β*=4.04 × 10^−4^, *P*<0.001), *APOE* ɛ4 (*β*=0.72, *P*<0.001) and the interaction between *APOE* ɛ4 and CSF Aβ38 (*β*=−3.40 × 10^−4^, *P*<0.001) were all significant, and CSF Aβ42 was a significant covariate (*β*=−0.00112, *P*<0.001). CSF Aβ38 (*P*=0.017), *APOE* ɛ4 (*P*<0.001) and the interaction between *APOE* ɛ4 and CSF Aβ38 (*P*=0.015) remained significant when removing CSF Aβ42 from the model. When predicting PET Aβ positivity using logistic regression, CSF Aβ38 (log odds=0.00332, *P*<0.001) and *APOE* ɛ4 (log odds=4.47, *P*=0.0075) were independent predictors and there was a tendency for significant interaction between *APOE* ɛ4 and CSF Aβ38 (*P*=0.061). Again, CSF Aβ42 was a significant covariate (log odds=−0.0119, *P*<0.001).

We also used a combination of CSF Aβ40 and CSF Aβ38 based on their molar amounts (CSF Aβ, mol l^−1^). Again, the results were very similar. When predicting continuous PET Aβ, the effects of CSF Aβ (*β*=3.70 × 10^8^, *P*<0.001), *APOE* ɛ4 (*β*=0.470, *P*<0.001) and the interaction between *APOE* ɛ4 and CSF Aβ (*β*=−2.20 × 10^8^, *P*=0.0030) were all significant predictors. CSF Aβ42 remained a significant covariate (*β*=−0.00120, *P*<0.001). CSF Aβ (*P*=0.0090), *APOE* ɛ4 (*P*<0.001) and the interaction between *APOE* ɛ4 and CSF Aβ (*P*=0.021) remained significant when removing CSF Aβ42 from the model. When predicting PET Aβ positivity using logistic regression, CSF Aβ (log odds=3.15 × 10^9^, *P*<0.001) was a significant predictor and CSF Aβ42 remained a significant covariate (log odds=−0.0129, *P*<0.001).

### CSF Aβ40 is highest in APOE ɛ4− PET Aβ+ subjects

**Figure 3:**
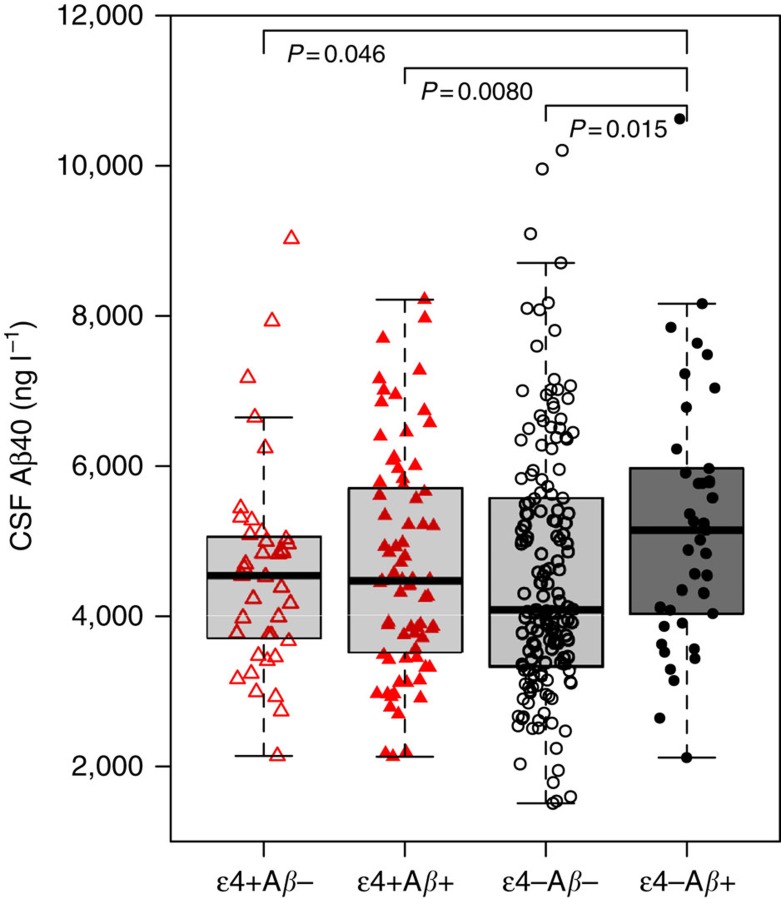
CSF Aβ40 in different combinations of PET Aβ and *APOE* ɛ4. Observed CSF Aβ40 for different combinations of *APOE* ɛ4 and PET Aβ positivity and negativity. The individual observations are overlaid on boxplots (thick lines are medians, box limits are 25th and 75th percentiles). CSF Aβ40 was significantly increased in the PET Aβ+ & *APOE* ɛ4− group compared with PET Aβ- & *APOE* ɛ4−, which was the reference category (*P*=0.015, using linear regression adjusted for age, sex, diagnosis and WML). No other group had significant different CSF Aβ40 compared with the PET Aβ- & *APOE* ɛ4− group (*P*=0.62–0.90). The groups were PET Aβ- & *APOE* ɛ4−, *N*=158; PET Aβ- & *APOE* ɛ4+, *N*=39; PET Aβ+ & *APOE* ɛ4−, *N*=41; and PET Aβ+ & *APOE* ɛ4+, *N*=75. The total *N*=313 for this analysis was smaller than the total study population (*N*=331) due to missing data for WML in 18 subjects (but the main results did not differ when WML was not included and the analysis was done on the whole study population). One data point is excluded from the graph for visual clarity (CSF Aβ40 14110, ng l^−1^, PET Aβ+ & *APOE* ɛ4−).

In a linear regression model with CSF Aβ40 as the dependent variable and a four level combination of PET Aβ and *APOE* as the independent variable, the overall highest CSF Aβ40 levels were seen in the PET Aβ+ & *APOE* ɛ4− group (*β*=732, *P*=0.015, compared with the reference category PET Aβ- & *APOE* ɛ4−, Fig. 3). PET Aβ+ & *APOE* ɛ4− subjects had 19% higher mean level of CSF Aβ40 (and 26% higher median level) compared with PET Aβ- & *APOE* ɛ4− subjects. The model was adjusted for WML (*β*=−18.8, *P*=0.00013), age (*β*=46.1, *P*=0.015), sex (female sex, *β*=−322, *P*=0.094) and diagnostic group (SCD, *β*=570, *P*=0.019; MCI, *β*=421, *P*=0.11). When also adjusting for CSF Aβ42 as a covariate the effect of PET Aβ & *APOE* ɛ4 was even stronger, with higher CSF Aβ40 in the PET Aβ+ & *APOE* ɛ4− group compared with PET Aβ- & *APOE* ɛ4− (*P*<0.001) and PET Aβ- & *APOE* ɛ4+ (*P*<0.001) but no significant difference compared with the PET Aβ+ & *APOE* ɛ4+ group (*P*=0.45).

## Discussion

We tested the hypothesis that biomarker surrogates of amyloidogenic APP processing (CSF Aβ40 and Aβ38) and *APOE* ɛ4 were independent predictors of brain Aβ fibril accumulation. In accordance with our hypotheses, CSF Aβ40 (and Aβ38 in a secondary analysis) and *APOE* ɛ4 were independent predictors of PET Aβ, and the effect of CSF Aβ40 was strongest in the *APOE* ɛ4-negative individuals. To our knowledge, this is the first study showing that increased Aβ production are associated with increased risk for sporadic brain Aβ accumulation. These novel results provide indirect evidence that brain Aβ pathology in humans may arise from two pathways, where one involves the *APOE* ɛ4 allele (likely causing reduced apoE4-mediated Aβ42 clearance), and the other involves increased amyloidogenic processing of APP. This may correspond to two pathways to sporadic AD, namely reduced clearance and increased production of Aβ peptides.

The amyloid cascade hypothesis postulates that Aβ pathology arises due to an imbalance between Aβ production and clearance^6^. It has been suggested that sporadic AD is mainly caused by poor clearance of peptides from the brain, whereas autosomal dominant AD is mainly caused by increased Aβ production, especially the Aβ42 variant. This is supported by a metabolic labelling study showing reduced Aβ clearance in sporadic AD dementia^14^, and studies showing increased amyloidogenic APP processing in early stages of autosomal dominant AD^10^. The main cause of reduced Aβ clearance in sporadic AD is likely *APOE* ɛ4, since the apoE4 protein isoform has reduced capacity to clear Aβ peptides compared with other apoE isoforms^8^, although it is possible that *APOE* ɛ4 may also contribute to increased AD risk by other mechanisms, for example, by affecting inflammation and neuronal repair^15^,^16^. However, one *APP* gene polymorphism which reduces Aβ production is associated with reduced risk of AD in the general population^17^, which provides genetic evidence that variations in APP processing may also affect the risk for sporadic AD.

The main limitation of this paper was that we used an indirect measure of APP processing, which was estimated by CSF Aβ40. The rationale for this approach was that Aβ40 is a major Aβ isoform produced by neurons^11^, which is not directly influenced by the presence of Aβ plaque pathology^12^, and is not influenced by *APOE* ɛ4-mediated impaired Aβ clearance. The later was demonstrated by our finding that there was no overall difference in CSF Aβ40 depending on *APOE* ɛ4 status (Fig. 1). Alterations in CSF Aβ40 are therefore more likely to reflect differences in amyloidogenic APP processing rather than differences in Aβ clearance. However, we acknowledge that there may be variations in APP processing that are not captured by CSF Aβ40. We also performed analyses using CSF Aβ38 (another highly expressed Aβ isoform) and a combination of CSF Aβ38 and CSF Aβ40 (based on their molar amounts), with very similar results as when using CSF Aβ40 alone, which support our findings. A more direct estimate of Aβ production may be done by metabolic labelling^14^, but such methods are liable to bias due to the longitudinal drift of CSF biomarkers during continuous CSF sampling that depends on sampling frequency and volume^18^. Another limitation is that there may be other factors affecting CSF Aβ40 besides variations in APP processing. For example, reduced CSF Aβ40 is associated with chronic WML^19^, and WML may also be associated with Aβ pathology (although this is more common in MCI^20^ and AD dementia^20^ than in non-demented people^21^). It is not clear if the association between CSF Aβ40 and WML is due to a direct link between Aβ production and WML or if lower CSF Aβ40 levels reflect reduced neuronal Aβ secretion due to decreased brain activity in the presence of WML. Importantly, our results remained significant when adjusting for WML. Theoretically, CSF Aβ40 could also be influenced by peripheral APP processing, but our results were stable when adjusting for plasma Aβ40, suggesting that the effects did not depend on peripheral APP processing. We did not measure all other possible factors besides increased amyloidogenic APP that may contribute to Aβ deposition in *APOE* ɛ4-negative subjects. For example, other AD risk genes (including *CLU* and *CR1*) may impact Aβ clearance in *APOE* ɛ4-negative subjects^22^. We included several different diagnostic groups, including a SCD group. We noted that the frequency of Aβ positivity in our SCD subjects (37%) was higher than in a recent large meta-analysis by Jansen *et al.*^3^ where ∼22% of SCD subjects were Aβ-positive, compared with ∼25% of CN subjects. The reason for this difference is not clear, but we noted that our SCD subjects were on average 6 years older than the Jansen subjects, which may contribute to higher frequency of Aβ pathology. Furthermore, all our SCD subjects were referred to specialized memory clinics because of cognitive symptoms, while some of the Jansen SCD subjects may have been seen at other health care facilities, opening for the possibility that they had less severe complaints than the SCD subjects in our study. Another recent study on PET Aβ positivity in memory clinic SCD subjects found that 57% of SCD subjects were Aβ-positive compared with 31% of CN, which more resembles the findings in our cohort^23^. Finally, we did not include an AD dementia group, since we know from a previous study that patients with severe AD dementia have lower CSF Aβ40 than patients with mild dementia (this may reflect reduced capacity to produce Aβ peptides as the disease progresses)^24^. Including an AD dementia group in this study would therefore risk confounding the relationship between CSF Aβ40 and PET Aβ.

Until now, there have been few attempts to examine the independent roles of *APOE* ɛ4 and APP processing in the development of brain Aβ pathology in non-demented subjects. Previous studies did not find correlations between CSF Aβ40 (or Aβ38) and PET Aβ (ref. 12). This is likely because they did not co-vary for *APOE* ɛ4 (and/or CSF Aβ42). Adjusting for *APOE* ɛ4 is important since the relationship between CSF Aβ40 and PET Aβ differs between *APOE* ɛ4-positive and -negative individuals. Furthermore, adjusting for *APOE* ɛ4 and CSF Aβ42 reduces the residual errors of the models, and some of this error may contribute to the variance of CSF Aβ40. Once this error is removed the correlation between CSF Aβ40 and PET Aβ can be better estimated. Our results add novel information and point to different possible pathways to Aβ pathology in humans. In sum, our results support the idea that sporadic Aβ accumulation may be partly associated with increased amyloidogenic APP production, especially in *APOE* ɛ4-negative subjects. The risk for sporadic AD may consequently depend on increased Aβ production, in addition to decreased Aβ clearance. This provides novel insight into disease mechanisms in AD and may be important for development of drugs targeting Aβ metabolism in early stages of AD.

## Methods

### Study population

The study population came from the Swedish BioFINDER study (Biomarkers For Identifying Neurodegenerative Disorders Early and Reliably). All available CN and non-demented patients with mild cognitive symptoms characterized as having SCD or MCI were included.

CN subject were originally enrolled from the population-based EPIC cohort. The inclusion criteria were: age ≥60 years old, MMSE 28-30, and fluent in Swedish. Exclusion criteria were: presence of subjective cognitive impairment, significant neurologic disease (for example, stroke, Parkinson's disease, multiple sclerosis), severe psychiatric disease (for example, severe depression or psychotic syndromes), dementia or MCI. All CN subjects underwent a thorough clinical assessment, including neurological, psychiatric and cognitive testing all performed by a medical doctor, in addition to MRI of the brain and relevant blood tests. The cognitive battery included MMSE, ADAS-cog (items 1–3), Trail Making A & B, Symbol Digit modalities, A quick test of cognitive speed, clock drawing, cube coping, letter S fluency and animals fluency. The medical doctor made a global assessment of whether the individual was cognitively healthy based on the test results in relation to education and age. All CN subjects had a Clinical Dementia Rating scale score of 0.

The SCD and MCI cases were recruited consecutively and were thoroughly assessed by physicians with special competence in dementia disorders. The inclusion criteria were: referred to a memory clinic due to possible cognitive impairment, not fulfilling the criteria for dementia, MMSE 24–30, age 60–80 years and, fluent in Swedish. The exclusion criteria were: cognitive impairment that without doubt could be explained by another condition (other than prodromal dementia); severe somatic disease; and refusing lumbar puncture or neuropsychological investigation. The classification in SCD or MCI was based on a neuropsychological battery and the clinical assessment of a senior neuropsychologist. The battery included tests for verbal ability (including A multiple-choice vocabulary test (SRB:1 (ref. 25) and semantic verbal fluency (Condition 2, D-KEFS (ref. 26), episodic memory (including Rey Auditory Verbal Learning Test (RAVLT (ref. 27), and Rey Complex Figure Test (RCFT (ref. 28)), visuospatial construction ability (including Block design (WAIS (ref. 29) and The copy trial of Rey Complex Figure Test), attention and executive functions (including Trail Making Test (D-KEFS (ref. 26) and Letter Verbal Fluency, Condition 1 (D-KEFS (ref. 26)). A senior neuropsychologist stratified all patients into those with SCD (no measurable cognitive deficits) or MCI according to the consensus criteria for MCI suggested by Petersen^30^.

The Regional Ethics Committee in Lund, Sweden, approved the study. All subjects gave written informed consent. For more details, see ref. 13 and www.biofinder.se.

### PET analysis

Brain Aβ was measured using ^18^F-flutemetamol PET (refs 31, 32). PET/CT scanning was conducted at two sites using the same type of scanner, a Philips Gemini TF 16. PET sum images from 90 to 110 min post injection were generated for the average uptake. MRI results were not used since this does not improve the quantification of ^18^F-flutemetamol data^33^. The images were analysed using the NeuroMarQ software provided by GE Healthcare. A volume of interest template was applied for nine bilateral regions (prefrontal, parietal, lateral temporal, medial temporal, sensorimotor, occipital, anterior cingulate and posterior cingulate/precuneus), combined in a global neocortical composite signal^33^. The SUVR was the global composite tracer uptake, normalized for the mean uptake in the cerebellar cortex (note that Thurfjell *et al.*^35^ found that ^18^F-flutemetamol PET SUVR had >98% concordance with visual reads independent of which reference region that was used). Most analyses in this study used continuous PET Aβ but when indicated a previously defined cutoff for Aβ positivity was used (>1.42 SUVR, based on mixture modelling analysis^13^).

### Cerebrospinal fluid analysis

All subjects underwent lumbar CSF sampling at baseline, following the Alzheimer's Association Flow Chart^35^. Samples were stored in 1 ml polypropylene tubes at −80 °C until analysis. CSF Aβ38, Aβ40 and Aβ42 were analysed by ELISA assays (EUROIMMUN AG, Lübeck, Germany). All analyses were performed by board-certified laboratory technicians who were blinded for clinical data and diagnoses. The CSF samples were randomized to avoid group bias. The analyses were performed during two different runs in batch, plates 1–20 using lot no. E140224AB for Aβ38, E130611AA for Aβ40 and E130607AA for Aβ42, and plates 21–24 using lot no. E150522BK for Aβ38, E150302A1 for Aβ40 and E150522AZ for Aβ42. Aliquots of two different pools of CSF were used as internal control samples, with CVs of 13.8% for Aβ38 for the first control with a mean of 695 pg ml^−1^ and 7.9% for Aβ38 for the second control with a mean of 1596, pg ml^−1^; 17.9% for Aβ40 for the first control with a mean of 1951, pg ml^−1^ and 11.1% for Aβ40 for the second control with a mean of 3992, pg ml^−1^; and 16.3% for Aβ42 for the first control with a mean of 227 pg ml^−1^ and 15.1% for Aβ42 for the second control with a mean of 216 pg ml^−1^. To assure consistency in levels between the two runs, 40 CSF samples from the first run were re-analysed in the second run.

### Plasma analysis

For plasma collection, blood was drawn into tubes containing EDTA as anticoagulant. After centrifugation (2000*g*, +4 °C, 10 min), plasma samples were aliquoted into polypropylene tubes and stored at −80 °C pending biochemical analyses. Plasma Aβ40 was analysed using Simoa immunoassay (Quanterix, Lexington, MA, USA).

### White matter lesions

All patients were examined using a single 3T MR scanner (Trio, Siemens). Automated segmentation of WML was performed using the Lesion Segmentation Tool implemented in SPM8 (http://www.applied-statistics.de/lst.html), generating a total WML volume. Before this, manual segmentation for reference of WML was performed on FLAIR images co-registered to the native MPRAGE in four MCI patients, with the segmented volume ranging from 0.5 to 106.3 ml; the resulting optimal κ based on the Dice coefficient was 0.4 (ref. 36) and was used in the subsequent automated segmentation for all participants.

### Statistical analysis

We tested correlations between CSF Aβ40 and PET Aβ in different regression models. The main model was a linear regression model where the dependent variable was PET Aβ and the independent variables were CSF Aβ40, *APOE* ɛ4 (dichotomous), and the interaction between CSF Aβ40 and *APOE* ɛ4. Second, we tested the correlation between clinically significant PET Aβ accumulation and CSF Aβ40 and *APOE* ɛ4 in a logistic regression model with PET Aβ positivity as the dependent variable. Third, we tested a linear regression model with CSF Aβ40 as the dependent variable and a four level combination of PET Aβ and *APOE* ɛ4 as the independent variable (PET Aβ- & *APOE* ɛ4−, PET Aβ- & *APOE* ɛ4+, PET Aβ+ & *APOE* ɛ4− and PET Aβ+ & *APOE* ɛ4+). All models were adjusted for age (years), sex and diagnostic group. We also adjusted for CSF Aβ42 to test if CSF Aβ40 was associated with PET Aβ beyond CSF Aβ42, and to reduce the residual error of the model, allowing a better estimate of the correlation between CSF Aβ40 and PET Aβ. We adjusted for WML (ml) except for when WML was clearly nonsignificant, as detailed in the results section. The primary analyses were done using CSF Aβ40, but we also performed analyses using CSF Aβ38 and a combination of CSF Aβ38 and Aβ40 (based on their molar weights, Aβ38: 4129.012 g mol^−1^; and Aβ40: 4327.148 g mol^−1^ (ref. 37). Statistical significance was determined at *P*<0.05. All analyses were done using R (v. 3.0.1, The R Foundation for Statistical Computing).

## Additional information

**How to cite this article:** Mattsson, N. *et al.* Increased amyloidogenic APP processing in *APOE* ɛ4-negative individuals with cerebral β-amyloidosis. *Nat. Commun.* 7:10918 doi: 10.1038/ncomms10918 (2016).

## Figures and Tables

**Table 1:** Demographics.

	**CN**	**SCD**	**MCI**	**All**	***P*** **value**
*N*	121	102	108	331	
Age (y)	73.7 (4.5)	70.2 (5.6)	71.2 (5.6)	71.8 (5.4)	<0.001
Sex (F)	63%	51%	40%	52%	0.0023
Education (y)	11.5 (3.28)	12.7 (3.28)	11.3 (3.38)	11.8 (3.39)	0.0038
MMSE (points)	29.0 (0.92)	28.5 (1.47)	27.2 (1.69)	28.3 (1.57)	<0.001
ADAS-cog, delayed recall (points)	2.1 (2.0)	3.3 (2.2)	6.4 (2.2)	3.9 (2.8)	<0.001
*APOE* ɛ4 (% +)	28%	42%	43%	37%	0.028
PET Aβ (% +)	19%	37%	61%	38%	<0.001
PET Aβ (SUVR)	1.30 (0.28)	1.41 (0.38)	1.70 (0.53)	1.46 (0.44)	<0.001
CSF Aβ38 (ng l^−1^)	1,742 (404)	1,722 (421)	1,686 (421)	1,718 (414)	0.51
CSF Aβ40 (ng l^−1^)	4,510 (1526)	4,893 (1852)	4,812 (1809)	4,727 (1728)	0.35
CSF Aβ42 (ng l^−1^)	538 (187)	584 (251)	475 (216)	532 (221)	0.0031

ADAS-cog, Alzheimer's Disease Assessment Scale-cognitive subscale; CN, cognitively normal; CSF, cerebrospinal fluid; MCI, mild cognitive impairment; MMSE, mini**-**mental state examination; PET, positron emission tomography; SCD, subjective cognitive decline; SUVR, standardized uptake value ratio.

Continuous data are mean (s.d.). *APOE* e4+ is defined as at least one e4 allele. PET Aβ positivity is defined as >1.42 SUVR (ref. 13). MMSE ranges from 0 to 30. ADAS-cog delayed recall ranges from 0 to 10 (word list learning test from the ADAS-cog battery, points indicate number of missed items). *P* values are for comparisons between diagnostic groups (using Kruskal–Wallis test for continuous variables and *X*^2^-test for categorical variables).

**Table 2:** Demographics by diagnostic group and *APOE* ɛ4.

	**CN**		**SCD**		**MCI**		**All**	
***N***	**121**		**102**		**108**		**331**	
	**APOE ɛ4−**	**APOE ɛ4+**	**APOE ɛ4−**	**APOE ɛ4+**	**APOE ɛ4−**	**APOE ɛ4+**	**APOE ɛ4−**	**APOE ɛ4+**
	87	34	59	43	61	47	207	124
Age (y)	73.5 (4.4)	74.1 (4.7)	70.1 (6.0)	70.3 (5.0)	71.0 (6.1)	71.5 (5.0)	71.8 (5.6)	71.8 (5.1)
Sex (F)	63%	62%	53%	49%	41%	38%	54%	48%
Education (y)	11.6 (3.3)	11.5 (3.3)	12.9 (3.5)	12.4 (2.9)	11.3 (3.4)	11.3 (3.3)	11.9 (3.5)	11.7 (3.2)
MMSE (points)	29.0 (0.97)	29.2 (0.78)	28.4 (1.55)	28.6 (1.35)	27.2 (1.75)	27.2 (1.62)	28.3 (1.58)	28.2 (1.57)
ADAS-cog, delayed recall (points)	2.2 (2.1)	1.7 (1.9)	3.2 (2.3)	3.5 (2.0)	6.0 (2.3)	6.9 (2.0)	3.6 (2.7)	4.3 (2.9)
PET Aβ (% +)	10%	41%	20%	61%	38%	91%	21%	67%
PET Aβ (SUVR)	1.22 (0.16)	1.48 (0.41)	1.27 (0.29)	1.59 (0.42)	1.51 (0.51)	1.95 (0.45)	1.32 (0.35)	1.70 (0.47)
CSF Aβ38 (ng l^−1^)	1,741 (420)	1,742 (365)	1,735 (457)	1,705 (367)	1,670 (436)	1,707 (405)	1,719 (435)	1,716 (380)
CSF Aβ40 (ng l^−1^)	4,573 (1,655)	4,348 (1,135)	5,076 (2,051)	4,643 (1,527)	4,792 (1,969)	4,838 (1,599)	4,781 (1,871)	4,636 (1,461)
CSF Aβ42 (ng l^−1^)	571 (179)	454 (181)	673 (240)	462 (213)	559 (211)	365 (168)	596 (212)	423 (192)

ADAS-cog, Alzheimer's Disease Assessment Scale-cognitive subscale; CN, cognitively normal; CSF, cerebrospinal fliuid; MCI, mild cognitive impairment; MMSE, mini-mental state examination; PET, or positron emission tomography; SCD, subjective cognitive decline; SUVR, standardized uptake value ratio.

Continuous data are mean (s.d.). *APOE* ɛ4+ is defined as at least one ɛ4 allele. PET Aβ positivity is defined as>1.42 SUVR (ref. 13).
